# Explore the Structural and Electronic Properties at the Organic/Organic Interfaces of Thiophene-Based Supramolecular Architectures

**DOI:** 10.3390/nano15080601

**Published:** 2025-04-14

**Authors:** Lixia Kang, Hui Lu, Shunze Xia, Xianfei Xu, Yao Tian, Zechao Yang

**Affiliations:** 1 School of Physics, Hangzhou Normal University, No. 2318, Yuhangtang Rd., Hangzhou 311121, China; kanglixia@hznu.edu.cn (L.K.); lu_hui2025@hznu.edu.cn (H.L.); xiashunze@126.com (S.X.); xfxu@stu.hznu.edu.cn (X.X.); tianyao@stu.hznu.edu.cn (Y.T.); 2 Institut für Experimentalphysik, Freie Universität Berlin, Arnimallee 14, 14195 Berlin, Germany

**Keywords:** scanning tunneling microscopy, organic/organic interface, molecular orbital alignment, charge transfer, oligothiophene

## Abstract

The structural and electronic properties at organic/organic interfaces determine the functionality of organic electronics. Here, we investigated the structural and electronic properties at interfaces between methyl-substituted dicyanovinyl-quinquethiophenes (DCV5T-Me_2_) and other electron acceptor molecules, namely fullerene (C_60_) and tetracyanoquinodimethane (TCNQ), by using low-temperature scanning tunneling microscopy/spectroscopy (STM/STS). Upon adsorption on Au(111), DCV5T-Me_2_ molecules self-assemble into compact islands at sub-monolayer coverage through hydrogen bonding and electrostatic interactions. A similar bonding configuration dominates in the second layer of a bilayer film, where DCV5T-Me_2_ possesses higher-lying LUMO (lowest unoccupied molecular orbital) and LUMO+1 in energy due to a decoupling effect. The co-deposition of DCV5T-Me_2_ and C_60_ does not result in ordered hybrid assemblies at the sub-monolayer coverage on Au(111). On the other hand, C_60_ molecules can self-assemble into ordered islands on top of the DCV5T-Me_2_ monolayer. The dI/dV spectra reveal that the LUMO of decoupled C_60_ is 400 mV lower in energy than the LUMO of decoupled DCV5T-Me_2_. This energy difference facilitates electron transfer from DCV5T-Me_2_ to C_60_. The co-deposition of DCV5T-Me_2_ and TCNQ leads to the formation of hybrid nanostructures. A tip-induced electric field can manipulate the charging and discharging of TCNQ by surrounding DCV5T-Me_2_, manifested as sharp peaks and dips in dI/dV spectra recorded over TCNQ.

## 1. Introduction

Molecular electronics, utilizing functional organic molecules as the primary building blocks for electronic components, offers a potential pathway for the next generation of electronic devices [1,2,3,4,5,6,7]. Enormous advances have been achieved in the last two decades. For example, organic-light emitting diodes (OLEDs), organic photovoltaic cells, and organic field-effect transistors have been constructed [1,2,5,6,7,8]. In this field, it is crucial to control the growth of organic thin films on metal surfaces and understand the related charge dynamics, which are mainly dominated by mechanisms like intermolecular interactions, metal-organic hybridization, charge transfer, and so on [4,8,9,10,11,12,13,14,15]. Within this scenario, the growth of mixed low-dimensional heteromolecular systems and the investigation of their electronic properties has boosted the actual application of organic molecules. The co-deposition of molecules with complementary structures and electronic properties could enhance the versatility of the supramolecular architectures [4,8]. On the other hand, interactions between different species of molecules increase the complexity of the system and make fully understanding the related charge dynamics challenging.

**Figure 1:**
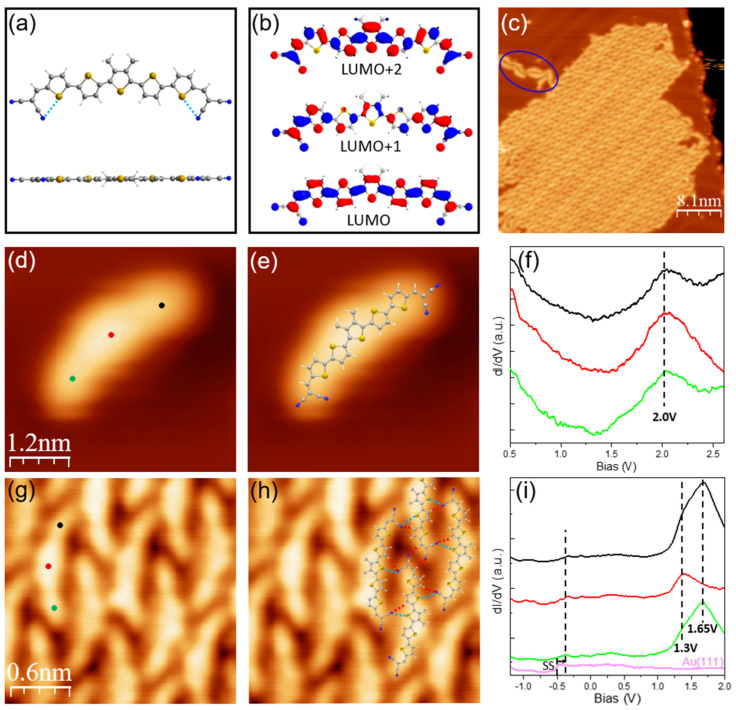
(**a**) Chemical structure and the DFT-optimized configuration of the isolated DCV5T-Me_2_ molecule. The color code is white: hydrogen, grey: carbon, blue: nitrogen and yellow: sulfur. The light blue dashed lines indicate electrostatic interaction between S and N atoms. (**b**) Calculated orbital structures of DCV5T-Me_2_ with the most stable configuration. (**c**) Overview STM image (I = 300 pA, V = 1.0 V) of DCV5T-Me_2_ deposited on room-temperature Au(111) surface. (**d**) High-resolution STM image (I = 1000 pA, V = 0.5 V) of a single DCV5T-Me_2_ on the surface. (**e**) STM image of the single DCV5T-Me_2_ with chemical structure model overlaid. (**f**) dI/dV spectra acquired with closed feedback (set point: I = 100 pA, V = 0.5 V) at different locations at a single molecule as indicated in the STM image in d. (**g**) High-resolution STM image (I = 200 pA, V = 0.4 V) of the molecular island. (**h**) The bonding structure of the molecular island. The red dashed lines indicate hydrogen bonds, while the light blue dashed lines indicate electrostatic interaction between S and N atoms. (**i**) dI/dV spectra acquired with open feedback (set point: I = 100 pA, V = −2 V) at different locations on the island as indicated in the STM image in g. The surface state is shifted up around 0.2 V due to the change in work function by the attachment of the organic layer. The “closed feedback” means active feedback loop, while “open feedback” means inactive feedback loop.

In this work, we studied the structural and electronic properties at interfaces between methyl-substituted dicyanovinyl-quinquethiophenes (DCV5T-Me_2_) and other electron acceptor molecules, namely fullerene (C_60_) and tetracyanoquinodimethane (TCNQ), by using low-temperature scanning tunneling microscopy/spectroscopy (STM/STS). The interest of DCV5T-Me_2_ originates from its internal electron acceptor-donor-acceptor structure. As shown in Figure 1a, a central electron-rich quinquethiophenes (5T) backbone is the electron donor, while two terminal electron-deficient dicyanovinyl (DCV) groups serve as acceptors [16,17,18,19,20,21,22,23,24]. The internal acceptor-donor-acceptor architecture modifies the electronic structure of oligothiophenes and makes it an interesting molecule for organic electronic devices. In particular, power conversion efficiencies over 10% have been realized in DCV5T-Me_2_-based heterojunction solar cells, where DCV5T-Me_2_ molecules transfer electrons to the C_60_ layer [16,17,18,19,20]. However, a microscopic study of the structural and electronic properties at the interfaces between DCV5T-Me_2_ and other electron acceptor molecules is still missing.

We investigated the DCV5T-Me_2_-based supramolecular architectures ranging from sub-monolayer up to bilayer coverage. First of all, the molecular orbital alignment of DCV5T-Me_2_ varies depending on the coverage. The LUMO and LUMO+1 of molecules in the first layer directly contacting the Au(111) metal surface show lower resonance energies compared with those in the second layer, which is attributed to the decoupling effect. Upon co-deposition of DCV5T-Me_2_ and C_60_ in the sub-monolayer coverage on Au(111), no hybrid structures can be formed. However, C_60_ molecules adopt the same orientation on top of the DCV5T-Me2 monolayer, forming ordered compact islands. The dI/dV spectra reveal that the LUMO of C_60_ growing on top of the DCV5T-Me_2_ monolayer is 400 mV lower in energy than the LUMO of decoupled DCV5T-Me_2_. This energy difference enables electron transfer from DCV5T-Me_2_ to C_60_ and makes them an organic charge-transfer complex. As for TCNQ, it can form hybrid nanostructures through intermolecular hydrogen bonding upon co-deposition with DCV5T-Me_2_ on Au(111). Moreover, DCV5T-Me_2_ donates electrons to TCNQ, forming an organic charge transfer system. Sharp peaks and dips appear in dI/dV spectra recorded over TCNQ, which is ascribed to the charging and discharging of TCNQ by a tip-induced electric field, respectively.

## 2. Methods

The experiments were carried out in a low-temperature STM working at 4.2 K and under ultra-high vacuum conditions (below 1 × 10^−10^ mbar). The Au(111) single crystal (from MaTecK) was cleaned by repeated cycles of Ar^+^ sputtering and subsequent annealing to around 810 K. DCV5T-Me_2_ molecules were deposited on Au(111) from a home-built molecular evaporator heated to 500 K. STM images were measured in the constant current mode and processed with the Nanotec WSxM software (version Beta 9.2) [25]. The dI/dV spectra were acquired with a lock-in amplifier with a frequency of 800 Hz and a modulation amplitude of 5 mV. DFT calculations were carried out by using the GAUSSIAN 03 W program package in the gas phase [26]. The 6-31G basis set and the B3LYP exchange–correlation function were used during the calculation. The DFT-calculated geometry in the gas phase is mainly used to estimate the molecular conformation upon adsorption on the surface for constructing bonding models in assemblies. The orbital energy levels derived from calculations in the gas phase are not utilized to directly compare with the corresponding experimental data.

## 3. Results and Discussion

The DCV5T-Me_2_ molecule has a central quinquethiophenes (5T) with the central thiophene ring substituted by two methyl (Me_2_) groups, which are symmetrically linked to two dicyanovinyl (DCV) functional groups. DFT calculations demonstrated that DCV5T-Me_2_ has several types of isomers in the gas phase, whose thiophene rings and DCV groups adopt different orientations. Among them, the slightly bent configuration with alternately oriented thiophene rings and the proximity of cyano groups and sulfur atoms from the neighboring thiophene rings has the lowest total energy (Figure 1a). Moreover, the structure of corresponding molecular orbitals (LUMO, LUMO+1, and LUMO+2) is also calculated. As shown in Figure 1b, the LUMO is delocalized over the entire molecule, whereas the LUMO+1 is mainly localized at the two sides, and the LUMO+2 is mainly distributed at the central part. In general, the DFT-optimized orbital structure in the gas phase can be utilized to compare with the measured orbital spatial distribution in experiments to estimate the effect of Au(111) upon adsorption.

### 3.1. Structural and Electronic Properties of DCV5T-Me_2_: From Sub-Monolayer to Bilayer

Upon deposition on Au(111) at sub-monolayer coverage, DCV5T-Me_2_ predominantly forms compact islands (Figure 1c). A few isolated DCV5T-Me_2_ monomers could also be observed on bare gold regions. Figure 1d shows a high-resolution STM image of the isolated DCV5T-Me_2_ monomer. One can identify that it adopts a bent configuration, perfectly fitting with the DFT-calculated configuration in the gas phase (Figure 1e). Figure 1g shows the high-resolution STM of a compact island, where DCV5TMe_2_ molecules have the same geometry as the individual molecule. Therefore, the DFT-calculated configuration could be utilized to construct the bonding model of islands. As shown in Figure 1h, one molecule points its cyano groups to the hydrogen and sulfur atoms of the neighboring molecules, which leads to intermolecular hydrogen bonds and electrostatic interactions, thereby assembling individual DCV5T-Me_2_ into compact islands. The intermolecular hydrogen bonding between dicyanovinyl-oligothiophenes has been imaged at atomic resolution by using low-temperature atomic force microscopy in our previous studies [22,27]. Moreover, the self-assembly of DCV5T-Me_2_ on Au(111) does not change the surface reconstruction, which indicates a relatively weak interaction between the gold surface and molecules [28]. It is worth noting that a few short chains (highlighted by blue oval in Figure 1c) are also observed on the surface. Those structures are formed through metal-ligand coordination between Au adatoms and the DCV groups, which has been reported in our previous study [21].

We performed STS measurements to investigate the electronic properties of DCV5TMe_2_ assemblies on Au(111). As a reference, STS was first recorded over the isolated monomer. As shown in Figure 1f, spectra at the center and at the DCV end-groups exhibit a clear peak at 2.0 V with similar intensity at the three locations, which is assigned to the LUMO-derived resonance. In contrast, the LUMO+1 with an onset of 2.5 V is only localized at the two ends. The extended and local character of the LUMO and LUMO+1 on Au(111) is in good agreement with the DFT-calculated orbital structure in the gas phase (Figure 1b), which indicates that DCV5T-Me_2_ maintains, to a large degree, their free-molecule character upon adsorption on Au(111) due to relatively weak surface-molecule interaction [21,22,23].

The dI/dV spectra obtained at molecules within the compact island show a peak at 1.3 V delocalized over the whole molecule and a higher-lying resonance at 1.65 V mainly localized at the molecular sides (Figure 1i). This spatial distribution allows us to assign these two peaks to the LUMO and LUMO+1, respectively. It means that the LUMO and LUMO+1 are shifted from 2.0 V to >2.5 V at the isolated molecule down to 1.3 V and 1.65 V in compact islands, respectively. We attribute this change in molecular orbital alignment in energy to the presence of intermolecular interactions. Here, it is mainly the intermolecular hydrogen bonds. It has been reported that the LUMO and LUMO+1 states of PTCDA molecules within different domains are different in energy [29,30]. The unoccupied molecular orbitals in a domain with stronger hydrogen bonds are shifted down 0.35 V overall compared with the states in a domain with relatively weaker bonds. The intermolecular hydrogen bonds facilitate the orbital overlap between molecules. Consequently, the spatial distribution of electrons within DCV5T-Me_2_ islands is more delocalized than electrons confined in one isolated DCV5T-Me_2_ molecule, thereby lowering their energy levels. This is a manifestation of the “particle in a box” in quantum mechanics [12,14,31,32,33].

Moreover, one can identify small step-like peaks around −0.3 V at the spectra in Figure 1i, which are from the gold surface state. The surface state penetrates the DCV5T-Me_2_ islands and is shifted up to −0.3 eV from −0.5 eV due to the increase in work function upon adsorption of organic molecules. This further confirms the physisorption and weak molecule-surface interaction on Au(111).

**Figure 2:**
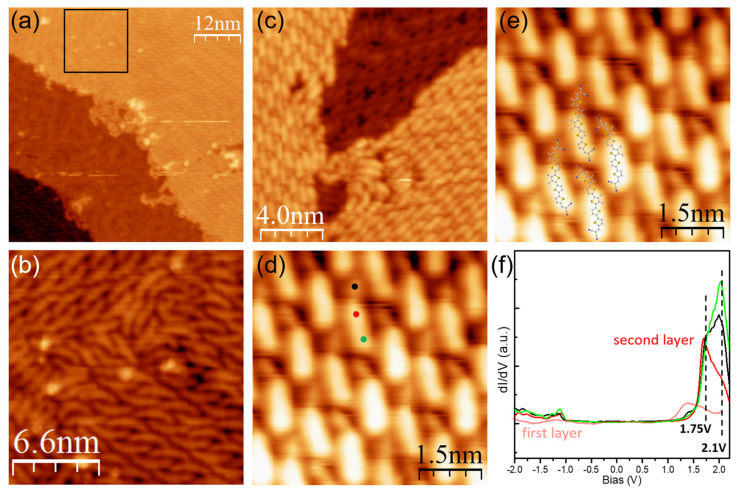
(**a**) Overview STM image (I = 100 pA, V = 0.5 V) of DCV5T-Me_2_ assemblies at monolayer coverage on Au(111). (**b**) High-resolution STM image (I = 100 pA, V = 0.5 V) of the area highlighted by the black square in a. (**c**) Overview STM image (I = 300 pA, V = 1.0 V) of DCV5T-Me_2_ assemblies at bilayer coverage on Au(111). (**d**) High-resolution STM image (I = 1000 pA, V = 0.5 V) of the second layer. (**e**) High-resolution STM image (I = 1000 pA, V = 0.5 V) of the second layer with molecular structure overlaid. (**f**) dI/dV spectra acquired with closed feedback (set point: I = 100 pA, V = 0.5 V) at different locations at the molecule in the second layer as indicated in the STM image in (**d**) [34]. The spectrum recorded with the same tip at the center of a molecule in the first layer is also shown as a reference.

Since the actual application of functional molecules in organic electronics is always related to monolayer or multiple-layer films, we further studied the properties of DCV5T-Me_2_ at higher coverages. Figure 2a shows a typical STM image of DCV5T-Me_2_ assemblies at monolayer coverage on Au(111). The corresponding high-resolution STM image (Figure 2b) reveals that the majority of molecules maintain the same configuration and bonding geometry as those in the sub-monolayer. In the meantime, a few DCV5T-Me_2_ molecules located between different domains deviate from the DFT-optimized configuration due to the limited space. DCV5T-Me_2_ starts to form islands on top of the first molecular layer when coverage is increased over one monolayer, as shown in Figure 2c. The second layer consists of the same unit cell as the first layer. The high-resolution STM image (Figure 2d,e) shows that molecules become asymmetric within the second layer, with a big lobe connected to a small lobe by a node. After overlapping the bonding models of two layers, we found that the position of molecules within the second layer is not commensurate with the first layer. It means that the growth of the second layer is not templated by the structure underneath. However, the asymmetric configuration should be attributed to the interactions between the two layers. It is expected that cyano groups and thiophene rings in the second layer rotate to interact with the thiophene rings and cyano groups in the first layer, respectively, due to molecular flexibility. This rotation would result in asymmetric geometry.

In order to inspect the evolution of molecular electronic properties with the layer growth, we performed spectroscopic measurements, as shown in Figure 2f. The molecule in the second layer possesses two resonances of 1.75 V and 2.1 V. The former is distributed at the center and the two sides of the molecule, while the latter is mainly localized at the side groups. This spatial distribution is the same as the distribution of LUMO and LUMO+1 of DCV5T-Me_2_ at the sub-monolayer coverage, which allows us to assign the resonances at 1.75 V and 2.1 V to the LUMO and LUMO+1 states, respectively. Moreover, the width of resonance peaks in the second layer is narrower than that in the first layer. The narrow width of the line shape and the higher lying in the energy of the LUMO and LUMO+1 in the second layer imply good decoupling of molecules within the second layer from the metal surface. However, the LUMO at the second layer is still lower in energy than that at the isolated molecule on the Au(111) surface. We attribute this to the existence of intermolecular interactions within the second layer and between the two layers, which shift down the molecular orbitals in energy as well.

### 3.2. Structural and Electronic Properties at DCV5T-Me_2_/C_60_ Interface

One important application of DCV5T-Me_2_ molecules in organic devices is serving as an electron donor in organic solar cells, where the fullerene (C_60_) molecules act as electron acceptors [18]. The molecular orbital alignments in energy at the DCV5T-Me_2_/C_60_ interface determine the charge transfer process and, hence, influence the performance of the device. Therefore, we investigated their interface properties at sub-molecular resolution with STM/STS when DCV5T-Me_2_ and C_60_ are co-deposited on Au(111).

**Figure 3:**
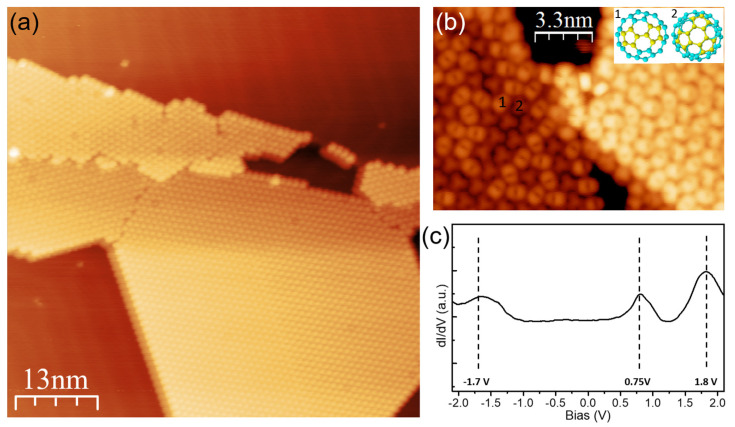
(**a**) Overview STM image (I = 200 pA, V = 0.5 V) of the self-assembly of C_60_ on Au(111) at room temperature. (**b**) High resolution STM image (I = 100 pA, V = 0.7 V) of the C_60_ islands. Inset: the model arrangement of C_60_. (**c**) dI/dV spectrum acquired with open feedback at the C_60_ molecule on Au(111) (set point: I = 110 pA, V = −2.2 V).

First, the structural and electronic properties of pure C_60_ assemblies on Au(111) are studied. Upon deposition on Au(111) at room temperature, C_60_ molecules self-assemble into two-dimensional islands with hexagonal symmetry, as shown in Figure 3a. The formation of compact islands is driven by weak van der Waals interactions with an average intermolecular distance of around 10 Å [35,36,37,38]. STS measurements recorded on C_60_ (Figure 3c) show three clear peaks around the Fermi energy, which are related to the molecular frontier orbitals. The peaks at 0.75 V and 1.8 V are assigned to the LUMO and LUMO+1 orbitals, while the resonance at −1.7 V is assigned to the HOMO (highest occupied molecular orbital). This results in a HOMO-LUMO gap of 2.45 eV, in good agreement with the values reported in the literature [35,36,37,38]. The high-resolution STM image recorded at the LUMO energy in Figure 3b reveals that the C_60_ molecules exhibit different symmetries within the island. The LUMO feature of C_60_ is dominated by the pentagons. Consequently, one can identify that C_60_ molecules mainly adopt two types of orientations upon adsorption on Au(111), which correspond to C_60_ molecules pointing their two or three pentagons out of the surface (inset of Figure 3b). The diversity of adsorption configuration indicates that the interaction between C_60_ and the underlying Au(111) surface is relatively weak.

**Figure 4:**
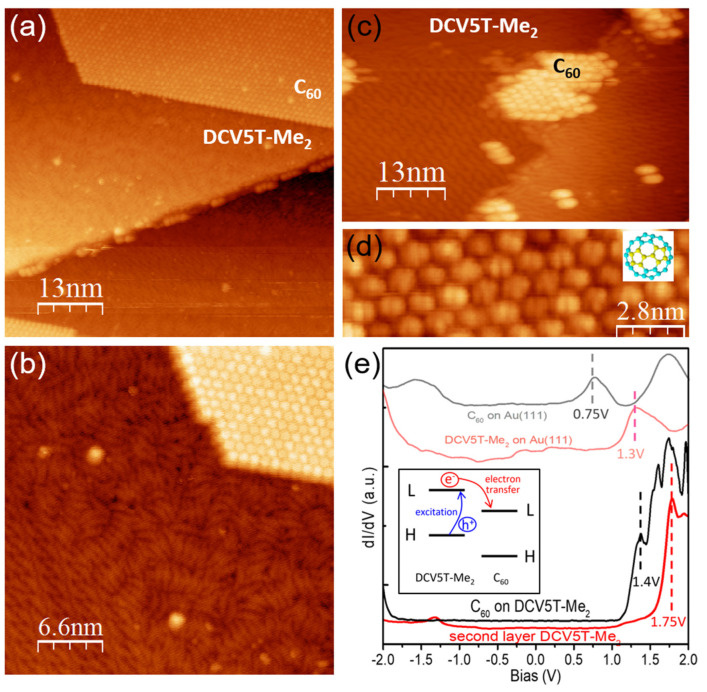
(**a**) Overview STM image (I = 100 pA, V = 0.5 V) of the co-deposition of C_60_ and DCV5T-Me_2_ on Au(111). (**b**) Zoomed-in STM image (I = 100 pA, V = 0.7 V) of the assembly. (**c**) STM image (I = 63 pA, V = 0.86 V) of deposition of monolayer of DCV5T-Me_2_ followed by evaporation of C_60_ at room temperature [34]. (**d**) High resolution STM image (I = 22 pA, V = 0.75 V) of C_60_ islands on top of the DCV5T-Me_2_ layer. (**e**) dI/dV spectra acquired with open feedback at C_60_ on Au(111) (gray: set point is I = 110 pA, V = −2.2 V), C_60_ on top of the DCV5T-Me_2_ monolayer (black: set point is I = 75 pA and V = −2.1 V), DCV5T-Me_2_ on Au(111) (pink: set point is I = 110 pA, V = −2 V) and the second layer DCV5T-Me_2_ on top of the DCV5T-Me_2_ monolayer (red: set point is I = 100 pA, V = 0.5 V) [34].

Figure 4a shows an overview STM image of the co-deposition of DCV5T-Me_2_ and C_60_ on Au(111) at monolayer coverage [34]. No hybrid assemblies can be formed with DCV5T-Me_2_ islands and C_60_ islands distributed separately on the surface. The high-resolution STM image in Figure 4b reveals that DCV5T-Me_2_ and C_60_ in the separate domains maintain, to a large degree, their packing structure in the sub-monolayer coverage. The domain separation is attributed to the rather strong intermolecular interactions between DCV5T-Me_2_ molecules. These results mean that DCV5T-Me_2_ and C_60_ cannot form an organic charge transfer complex at low coverage.

Another approach to mixing them is growing hybrid films with alternate DCV5T-Me_2_ and C_60_ layers. Therefore, we deposited C_60_ on top of a saturated DCV5T-Me_2_ monolayer on Au(111). As shown in Figure 4c, typical hexagonal islands of C_60_ are formed on the DCV5T-Me_2_ film. A high-resolution STM image reveals that C_60_ adopts only one type of orientation with a two-fold symmetry axis (Figure 4d). This homogeneous orientation could be ascribed to the π-π interaction between C_60_ and DCV5T-Me_2_, presumably stronger than the interaction between the C_60_ and Au(111) surface.

The dI/dV spectra show that C_60_ on the DCV5T-Me_2_ monolayer possesses a LUMO orbital at 1.4 V (black in Figure 4e), which is higher in energy compared with the LUMO of C_60_ directly contacting Au(111) (gray in Figure 4e) [34]. The higher-lying LUMO of C_60_ is attributed to the decoupling effect of the organic film of DCV5T-Me_2_ [39]. However, the far-lying of C_60_ LUMO from the Fermi level in energy indicates that no electrons are transferred from DCV5T-Me_2_ in the first layer to the decoupled C_60_. This can be interpreted from the point of view of energy alignment at their interface. The LUMO of the decoupled C_60_ is around 1.4 V (black in Figure 4e), which is higher than the LUMO of DCV5T-Me_2_ (1.3 V) on Au(111) (pink in Figure 4e). This orbital energy alignment inhibits electron transfer from DCV5T-Me_2_ to C_60_ because electrons cannot move from a lower energy level to a higher one. On the other hand, the LUMO of decoupled C_60_ is 350 mV lower in energy than the LUMO of decoupled DCV5T-Me_2_ (red in Figure 4e). As sketched in the inset of Figure 4e, this energy difference can facilitate the electron transfer from DCV5T-Me_2_ to C_60_. It tells us that DCV5T-Me_2_ and C_60_ are a good combination to form P-N junctions for organic electronics when decoupled from the metal surface.

### 3.3. Structural and Electronic Properties at DCV5T-Me_2_/TCNQ Interface

**Figure 5:**
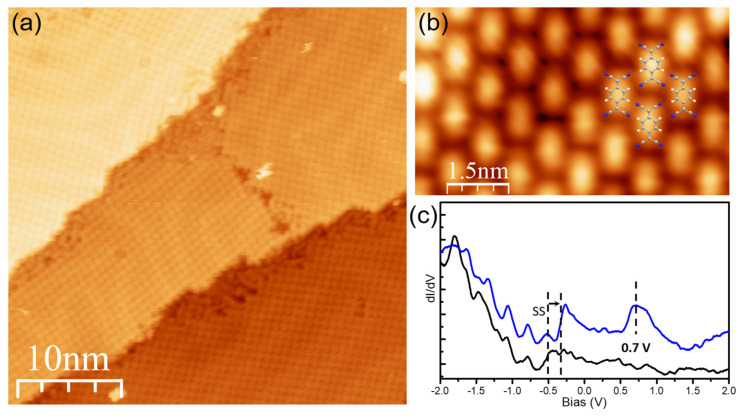
(**a**) Overview STM image (I = 100 pA, V = 0.5 V) of the self-assembly of TCNQ molecules on Au(111). (**b**) High-resolution STM image (I = 1000 pA, V = 0.2V) of the assembly with the molecular model overlaid [34]. (**c**) dI/dV spectra acquired with open feedback at TCNQ on Au(111) (set point I = 100 pA, V = −2.0 V).

One reason for the domain separation of DCV5T-Me_2_ and C_60_ at low coverage could be the spherical shape of C_60_, which weakens its interaction with the two-dimensional DCV5T-Me_2_. Tetracyanoquinodimethane (TCNQ) is a planar molecule (Figure 5b) and is a typical electron acceptor [40,41,42]. Therefore, we further used the planar TCNQ molecules to mix with DCV5T-Me_2_ in order to obtain a two-dimensional charge transfer complex.

Figure 5a shows an overview STM image of the self-assembly of TCNQ molecules on Au(111) at room temperature [34]. One can find that TCNQ forms highly ordered domains extending over hundreds of nanometers. The STM image further shows that the herringbone reconstruction of the Au(111) surface is unperturbed upon being covered by the TCNQ assemblies. This is a characteristic fingerprint of weak adsorption of TCNQ on Au(111). The high-resolution STM image reveals that the molecular structure has a rhombic unit cell (Figure 5b), where one TCNQ points its cyano groups to the -CH of its neighbor. This packing geometry results in a saturated C≡N· · · H-C hydrogen bond network [28]. The dI/dV spectrum recorded at TCNQ exhibits an unoccupied resonance at 0.7 V (Figure 5c), which is assigned to the molecular LUMO orbital. The location of LUMO well above the Fermi level indicates no electron transferred from Au(111) to TCNQ, despite the strong acceptor character of TCNQ. Note that the Au(111) surface state penetrates the molecule and is shifted up by 150 meV towards the Fermi level due to the increase in work function upon molecular adsorption.

**Figure 6:**
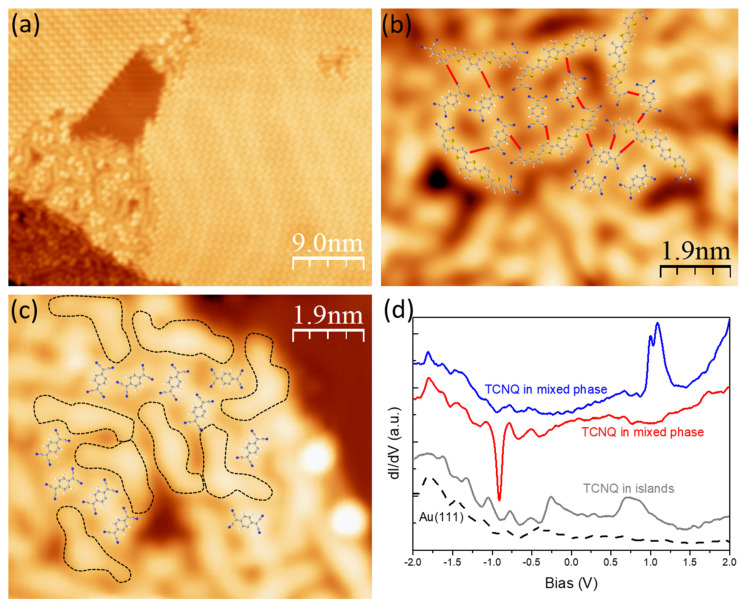
Mixing DCV5T-Me_2_ with TCNQ on Au(111) [34]. (**a**) STM overview (I = 63 pA, V = 0.8 V) of the co-deposition of DCV5T-Me_2_ and TCNQ on cold sample followed by annealing to 373 K. Large TCNQ islands and disordered mixture domains are formed. (**b**,**c**) High-resolution STM images (**b**: I = 53 pA, V = 1.12 V, **c**: I = 53 pA, V = 0.71 V) of the mixture domains with molecular model superimposed. Red lines in **b** indicate hydrogen bonds between DCV5T-Me_2_ and TCNQ. Black dash lines in c sketch the shape of DCV5T-Me_2_. (**d**) dI/dV spectra acquired with open feedback (set point: I = 130 pA, V = −2 V) at TCNQ in mixed-phase (blue and red) and ordered TCNQ islands (gray).

Figure 6a shows an overview STM image of the co-deposition of TCNQ and DCV5T-Me_2_ on Au(111). Compact TCNQ domains and disordered hybrid structures consisting of TCNQ and DCV5T-Me_2_ are formed [34]. A High-resolution STM image (Figure 6b) of the mixed phase reveals that TCNQ is randomly embedded amongst the DCV5T-Me_2_ molecules. By superimposing the molecular models on the STM image, it is determined that intermolecular hydrogen bonds (red lines in Figure 6b) between cyano ligands and -CH groups are formed. Although these hydrogen bonds are not strong enough to drive the assembly of an ordered hybrid structure, they are comparable in strength with the bonding between two DCV5T-Me2, enabling the growth of a hybrid structure. During mixing, some DCV5T-Me_2_ molecules adopt asymmetric configurations to optimize the bonding with adjacent TCQN molecules, as shown in Figure 6c. All these results demonstrate that a mixed structure can be obtained by co-deposition of DCV5T-Me_2_ and TCNQ on Au(111) [41].

To inspect the influence of interactions between DCV5T-Me_2_ and TCNQ on their electronic properties, we performed STS measurements. As shown in Figure 6d, spectra at TCNQ molecules in the mixed phase exhibit either a sharp peak (blue) around +1 V or a sharp deep (red) around −1 V, while the LUMO-derived resonance at +0.7 V (gray) in the TCQN islands is absent there. As reported on a TCNQ-based charge transfer complex, the sharp peak and deep are the fingerprints of discharging and charging electrons over single TCNQ molecules, respectively [40]. The electrons transferred from DCV5T-Me_2_ occupy the LUMO of TCNQ. The occupied TCNQ^−^ LUMO is shifted up in energy when a positive bias is applied to the TCNQ by the tip. With the bias ramping up to a certain value during STS measurement, the energy level of the TCNQ^−^ LUMO will be higher than the surface Fermi level, which induces the extra electrons transferred from TCNQ to the metal surface. This sudden discharging results in an increase in the tunneling current and, consequently, a sharp peak in the dI/dV spectrum. The dip is the result of the charging process of TCNQ by DCV5T-Me_2_ at negative bias, which can be explained by the same mechanism. The intensity and energy position of the peak and deep vary with the STM tip positions located over different TCNQ molecules because of the change in chemical environments for each TCNQ molecule in the disordered structure. Nevertheless, these features definitely demonstrate that electrons are transferred from DCV5T-Me_2_ to TCNQ at their interface. Therefore, in addition to C_60_, TCNQ could be another option to combine with DCV5T-Me_2_ molecules for building organic electronic devices. The stronger interaction between TCNQ and DCV5T-Me_2_ than that between DCV5T-Me_2_ and C_60_, may result in better performance of the device.

## 4. Conclusions

We explored the structural and electronic properties at the organic/organic interfaces between DCV5T-Me_2_, C_60_, and TCNQ at the sub-molecular level on Au(111). DCV5T-Me_2_ self-assembles into compact organic islands with coverage ranging from the sub-monolayer to the bilayer on Au(111). Its LUMO and LUMO+1 evolve with the change in coupling strength with the metal surface. DCV5T-Me_2_ in the second layer has higher-lying unoccupied orbitals in energy than molecules in the first layer due to the decoupling effect. The co-deposition of DCV5T-Me_2_ and C_60_ does not result in hybrid structures at the sub-monolayer coverage due to the three-dimensional shape of C_60_. On the other hand, C_60_ molecules can self-assemble into order islands over the DCV5T-Me_2_ monolayer. In this hybrid-layered system, the LUMO of decoupled C_60_ is 400 mV lower in energy than the LUMO of decoupled DCV5T-Me_2_, which enables electron transfer from DCV5T-Me_2_ to C_60_. Co-deposition of DCV5T-Me_2_ and TCNQ leads to the formation of hybrid nanostructures at sub-monolayer coverage. The tip-induced electric field can manipulate the charging and discharging of TCNQ by surrounding DCV5T-Me_2_, manifested as sharp peaks and dips in dI/dV spectra recorded over TCNQ. Our results provide valuable information for the application of dicyanovinylene-substituted oligothiophenes in organic electronics based on the charge transfer effect.

## Data Availability

Data are contained within the article.

## References

[1] Gupta R., Fereiro J.A., Bayat A., Pritam A., Zharnikov M., Mondal P.C. (2023). Nanoscale molecular rectifiers. Nat. Rev. Chem..

[2] Heath J.R., Ratner M.A. (2003). Molecular Electronics. Phys. Today.

[3] Li T., Bandari V.K., Schmidt O.G. (2023). Molecular Electronics: Creating and Bridging Molecular Junctions and Promoting Its Commercialization. Adv. Mater..

[4] Li X., Xu Z., Bu D., Cai J., Chen H., Chen Q., Chen T., Cheng F., Chi L., Dong W. (2024). Recent progress on surface chemistry II: Property and characterization. Chin. Chem. Lett..

[5] Xiang D., Wang X., Jia C., Lee T., Guo X. (2016). Molecular-Scale Electronics: From Concept to Function. Chem. Rev..

[6] Xin N., Guan J.X., Zhou C.G., Chen X.J.N., Gu C.H., Li Y., Ratner M.A., Nitzan A., Stoddart J.F., Guo X.F. (2019). Concepts in the design and engineering of single-molecule electronic devices. Nat. Rev. Phys..

[7] Zhang J.L., Zhong J.Q., Lin J.D., Hu W.P., Wu K., Xu G.Q., Wee A.T.S., Chen W. (2015). Towards single molecule switches. Chem. Soc. Rev..

[8] Stone I., Starr R.L., Zang Y.P., Nuckolls C., Steigerwald M.L., Lambert T.H., Roy X., Venkataraman L. (2021). A single-molecule blueprint for synthesis. Nat. Rev. Chem..

[9] Albrecht F., Neu M., Quest C., Swart I., Repp J. (2013). Formation and characterization of a molecule-metal-molecule bridge in real space. J. Am. Chem. Soc..

[10] Björk J., Matena M., Dyer M.S., Enache M., Lobo-Checa J., Gade L.H., Jung T.A., Stöhr M., Persson M. (2010). STM fingerprint of molecule-adatom interactions in a self-assembled metal-organic surface coordination network on Cu(111). Phys. Chem. Chem. Phys..

[11] Calzolari A., Ruini A., Catellani A. (2011). Anchor group versus conjugation: Toward the gap-state engineering of functionalized ZnO(1010) surface for optoelectronic applications. J. Am. Chem. Soc..

[12] Colazzo L., Mohammed M.S.G., Gallardo A., Abd El-Fattah Z.M., Pomposo J.A., Jelinek P., de Oteyza D.G. (2019). Controlling the stereospecific bonding motif of Au-thiolate links. Nanoscale.

[13] Li S.W., Zhang R.X., Kang L.X., Li D.Y., Xie Y.L., Wang C.X., Liu P.N. (2021). Steering Metal-Organic Network Structures through Conformations and Configurations on Surfaces. ACS Nano.

[14] Piquero-Zulaica I., Lobo-Checa J., Abd El-Fattah Z.M., Ortega J.E., Klappenberger F., Auwärter W., Barth J.V. (2022). Engineering quantum states and electronic landscapes through surface molecular nanoarchitectures. Rev. Mod. Phys..

[15] Zhao X.Y., Miao X.R. (2024). Surface-supported metal-organic frameworks with geometric topological diversity via scanning tunneling microscopy. Iscience.

[16] Fitzner R., Reinold E., Mishra A., Mena-Osteritz E., Ziehlke H., Körner C., Leo K., Riede M., Weil M., Tsaryova O. (2011). Dicyanovinyl-Substituted Oligothiophenes: Structure-Property Relationships and Application in Vacuum-Processed Small-Molecule Organic Solar Cells. Adv. Funct. Mater..

[17] Haid S., Mishra A., Uhrich C., Pfeiffer M., Bäuerle P. (2011). Dicyanovinylene-Substituted Selenophene-Thiophene Co-oligomers for Small-Molecule Organic Solar Cells. Chem. Mater..

[18] Mishra A., Bauerle P. (2012). Small molecule organic semiconductors on the move: Promises for future solar energy technology. Angew. Chem. Int. Ed. Engl..

[19] Mishra A., Uhrich C., Reinold E., Pfeiffer M., Bäuerle P. (2011). Synthesis and Characterization of Acceptor-Substituted Oligothiophenes for Solar Cell Applications. Adv. Energy Mater..

[20] Schrader M., Fitzner R., Hein M., Elschner C., Baumeier B., Leo K., Riede M., Bauerle P., Andrienko D. (2012). Comparative study of microscopic charge dynamics in crystalline acceptor-substituted oligothiophenes. J. Am. Chem. Soc..

[21] Yang Z., Corso M., Robles R., Lotze C., Fitzner R., Mena-Osteritz E., Bauerle P., Franke K.J., Pascual J.I. (2014). Orbital redistribution in molecular nanostructures mediated by metal-organic bonds. ACS Nano.

[22] Yang Z.C., Lotze C., Corso M., Baum S., Franke K.J., Pascual J.I. (2019). Direct Imaging of the Induced-Fit Effect in Molecular Self-Assembly. Small.

[23] Yang Z.C., Lotze C., Franke K.J., Pascual J.I. (2021). Metal-Organic Superlattices Induced by Long-Range Repulsive Interactions on a Metal Surface. J. Phys. Chem. C.

[24] Xia S.Z., Xu X.F., Tian Y., Zhong J.Q., Yang Z.C. (2025). Protected electronic structures of unoccupied molecular orbitals in metal-organic contacts. Appl. Surf. Sci..

[25] Horcas I., Fernandez R., Gomez-Rodriguez J.M., Colchero J., Gomez-Herrero J., Baro A.M. (2007). WSXM: A software for scanning probe microscopy and a tool for nanotechnology. Rev. Sci. Instrum..

[26] Frisch M.J., Trucks G.W., Schlegel H.B., Scuseria G.E., Robb M.A., Cheeseman J.R., Montgomery J.A., Vreven T., Kudin K.N., Burant J.C. (2004). Gaussian 03, Revision C.02.

[27] Bogner L., Yang Z., Corso M., Fitzner R., Bäuerle P., Franke K.J., Pascual J.I., Tegeder P. (2015). Electronic structure and excited state dynamics in a dicyanovinyl-substituted oligothiophene on Au(111). Phys. Chem. Chem. Phys..

[28] Torrente I.F., Franke K.J., Pascual J.I. (2008). Structure and electronic configuration of tetracyanoquinodimethane layers on a Au(111) surface. Int. J. Mass. Spectrom..

[29] Gonzalez-Lakunza N., Cañas-Ventura M.E., Ruffieux P., Rieger R., Müllen K., Fasel R., Arnau A. (2009). Hydrogen-Bonding Fingerprints in Electronic States of Two-Dimensional Supramolecular Assemblies. Chemphyschem.

[30] Kröger J., Jensen H., Berndt R., Rurali R., Lorente N. (2007). Molecular orbital shift of perylenetetracarboxylic-dianhydride on gold. Chem. Phys. Lett..

[31] Piquero-Zulaica I., Li J., Abd El-Fattah Z.M., Solianyk L., Gallardo I., Monjas L., Hirsch A.K.H., Arnau A., Ortega J.E., Stohr M. (2019). Surface state tunable energy and mass renormalization from homothetic quantum dot arrays. Nanoscale.

[32] Klappenberger F., Kuhne D., Krenner W., Silanes I., Arnau A., Garcia de Abajo F.J., Klyatskaya S., Ruben M., Barth J.V. (2011). Tunable quantum dot arrays formed from self-assembled metal-organic networks. Phys. Rev. Lett..

[33] Wang H., Zhang X., Jiang Z., Wang Y., Hou S. (2018). Electronic confining effects in Sierpiński triangle fractals. Phys. Rev. B.

[34] Yang Z. (2014). Structural and Electronic Properties of Thiophene-based Supramolecular Architectures on Metal Surfaces. Ph.D. Thesis.

[35] Fartash A. (1996). Dielectric properties of orientationally ordered/disordered C60(111) films. Phys. Rev. B.

[36] Schull G., Berndt R. (2007). Orientationally ordered (7 × 7) superstructure of C60 on AU(111). Phys. Rev. Lett..

[37] Yuan L.F., Yang J., Wang H., Zeng C., Li Q., Wang B., Hou J.G., Zhu Q., Chen D.M. (2003). Low-temperature orientationally ordered structures of two-dimensional C60. J. Am. Chem. Soc..

[38] Chen W., Zhang H., Huang H., Chen L., Wee A.T. (2008). Orientationally ordered C60 on p-sexiphenyl nanostripes on Ag111. ACS Nano.

[39] Franke K.J., Schulze G., Henningsen N., Fernandez-Torrente I., Pascual J.I., Zarwell S., Ruck-Braun K., Cobian M., Lorente N. (2008). Reducing the molecule-substrate coupling in C60-based nanostructures by molecular interactions. Phys. Rev. Lett..

[40] Fernandez-Torrente I., Kreikemeyer-Lorenzo D., Strozecka A., Franke K.J., Pascual J.I. (2012). Gating the charge state of single molecules by local electric fields. Phys. Rev. Lett..

[41] Jackel F., Perera U.G., Iancu V., Braun K.F., Koch N., Rabe J.P., Hla S.W. (2008). Investigating molecular charge transfer complexes with a low temperature scanning tunneling microscope. Phys. Rev. Lett..

[42] Fernandez-Torrente I., Franke K.J., Pascual J.I. (2008). Vibrational Kondo effect in pure organic charge-transfer assemblies. Phys. Rev. Lett..

